# NITRATE-CIN Study: Protocol of a Randomized (1:1) Single-Center, UK, Double-Blind Placebo-Controlled Trial Testing the Effect of Inorganic Nitrate on Contrast-Induced Nephropathy in Patients Undergoing Coronary Angiography for Acute Coronary Syndromes

**DOI:** 10.1177/1074248421000520

**Published:** 2021-03-25

**Authors:** Anne-Marie Beirne, Oliver Mitchelmore, Susana Palma, Mervyn Andiapen, Krishnaraj S. Rathod, Victoria Hammond, Anna Bellin, Jackie Cooper, Paul Wright, Sotiris Antoniou, Muhammad Magdi Yaqoob, Huseyin Naci, Anthony Mathur, Amrita Ahluwalia, Daniel A. Jones

**Affiliations:** 1 Centre for Cardiovascular Medicine and Devices, William Harvey Research Institute, 560754 Queen Mary University of London, United Kingdom; 2 Barts Interventional Group, Barts Heart Centre, Barts Health NHS Trust, London, United Kingdom; 3 Barts Cardiovascular Clinical Trials Unit, 560754 Queen Mary University of London, London, United Kingdom; 4 Department of Pharmacy, 560754 Barts Heart Centre, London, United Kingdom; 5 Department of Nephrology, 9744 Barts Health NHS Trust, London, United Kingdom; 6 Department of Health Policy, 4905 London School of Economics, London, United Kingdom

**Keywords:** contrast-induced nephropathy, acute coronary syndrome, percutaneous coronary intervention, inorganic nitrate, renal biomarkers

## Abstract

**Background:**

Contrast-induced nephropathy (CIN), an acute kidney injury resulting from the administration of intravascular iodinated contrast media, is a significant cause of morbidity/mortality following coronary angiographic procedures in high-risk patients. Despite preventative measures intended to mitigate the risk of CIN, there remains a need for novel effective treatments. Evidence suggests that delivery of nitric oxide (NO) through chemical reduction of inorganic nitrate to NO may offer a novel therapeutic strategy to reduce CIN and thus preserve long term renal function.

**Design:**

The NITRATE-CIN trial is a single-center, randomized, double-blind placebo-controlled trial, which plans to recruit 640 patients presenting with acute coronary syndromes (ACS) who are at risk of CIN. Patients will be randomized to either inorganic nitrate therapy (capsules containing 12 mmol KNO_3_) or placebo capsules containing potassium chloride (KCl) daily for 5 days. The primary endpoint is development of CIN using the Kidney Disease Improving Global Outcomes (KDIGO) criteria. A key secondary endpoint is renal function over a 3-month follow-up period. Additional secondary endpoints include serum renal biomarkers (e.g. neutrophil gelatinase-associated lipocalin) at 6 h, 48 h and 3 months following administration of contrast. Cost-effectiveness of inorganic nitrate therapy will also be evaluated.

**Summary:**

This study is designed to investigate the hypothesis that inorganic nitrate treatment decreases the rate of CIN as part of semi-emergent coronary angiography for ACS. Inorganic nitrate is a simple and easy to administer intervention that may prove useful in prevention of CIN in at-risk patients undergoing coronary angiographic procedures.

## Background

Contrast-induced nephropathy (CIN) refers to a form of acute kidney injury (AKI) that occurs after parenteral administration of radiopaque contrast agent. It is the 3rd most common cause of hospital-acquired renal failure, after decreased renal perfusion and use of nephrotoxic medications^1^ and remains a major unmet clinical need. CIN is associated with a prolonged hospital stay, higher hospitalization costs, and a significant increase in morbidity and mortality.^2,3^

CIN is commonly seen following procedures for acute coronary syndromes (ACS) with rates 3-5 times higher compared to elective procedures, with reported incidence ranging from 5%-55%, dependent upon the criteria used for diagnosis, the clinical setting and the investigated population.^4-8^ Additionally, emerging evidence suggests that 15%-20% of patients who do not fulfill the current serum-creatinine-based consensus criteria for AKI are nevertheless likely to have acute tubular damage (that can be identified by raised serum biomarkers of renal injury including neutrophil gelatinase-associated lipocalin (NGAL), cystatin-C, and interleukin-18 (IL-18)) and may also benefit from therapies targeting CIN.^9^

Whist the pathophysiology of CIN is not yet fully understood, an important mechanism thought to underlie the condition is the production of reactive oxygen species (ROS) and consequent vasoconstrictive renal hypoxic injury.^10,11^ One of the likely mechanisms of this is decreased levels of nitric oxide (NO) as a consequence of NO synthase dysfunction and NO scavenging.^12,13^ Thus, strategies that offer a mode of replacement of this “lost” NO represent an approach that may confer therapeutic benefit.

A potential solution for elevating endogenous NO levels lies in the non-canonical pathway for NO generation,^14^ whereby inorganic nitrate (NO_3_^−^) undergoes reduction to nitrite (NO_2_^−^) and then NO in the body. Furthermore, supplementation of NO_3_^−^ through the diet (e.g. green leafy vegetables or beetroot) or via administration of the anion in the form of a salt,^15^ delivers NO_2_^−^ into the circulation that is then reduced to NO at sites of need through the activity of specific NO_2_^−^ reductases.^14^

Limited data^16^ exists assessing the effect of NO on CIN, especially in patients undergoing percutaneous coronary intervention (PCI). In one small study, post-procedural renal function was compared between 112 patients who received an organic nitrate prior to PCI and 87 who did not. In those receiving the organic nitrate, 15.2% developed renal impairment compared to 29.9% in those who did not (OR: 0.42, 95% CI 0.21-0.84, *P* = .014). Multivariate logistic regression demonstrated use of organic nitrate was independently correlated with a reduction in the development of CIN (OR = 0.33, 95% CI 0.16-0.71, *P* = .004). In the NITRITE-AMI study^17^ we showed that a single bolus of intracoronary NO_2_^−^ given during primary PCI for ST-elevation MI led to a significant rise in circulating NO_2_^−^ levels. Additionally, during the first 48 hours after reperfusion, 5 patients (12.5%) in the control group developed CIN, which contrasted with only 1 patient (2.5%) in the NO_2_^−^ treated group. Further analysis indicated a statistically significant difference in the change in creatinine from baseline to 48 hours between the 2 groups (NO_2_^−^ group: −4.4 ± 3.6 µmol/L vs Placebo: 9.5 ± 3.7 µmol/L, *P* = .015), and lower levels of NGAL at 4 hours (105.7 ± 46.7 ng/mL vs 76.7 ± 31.9 ng/mL, *P =* .0018) and Cystatin C at 24 hours (1093 ± 324.1 pg/mL vs 950 ± 182.4 pg/mL, *P* ≤ .0001) in the NO_2_^−^ treated group suggesting reduced rates of subclinical CIN.^17^ This small phase II study and the pre-clinical data highlight the potential use of inorganic NO_3_^−^ in this group.

While these studies are encouraging there are limitations with either approach. It is well accepted that continuous/repeated administration of an organic nitrate is of limited benefit due to the development of tolerance^18^ and that raising circulating levels of inorganic NO_2_^−^ with a NO_2_^−^ salt is impacted by issues of toxicity.^19^ We hypothesize that a safe NO-delivery approach, without issues of tolerance, is inorganic NO_3_^−^; and this forms the basis for the study described below.

## Methods

### Study Hypothesis

The primary hypothesis to be tested is whether inorganic NO_3_^−^ therapy decreases the rate of CIN arising from the administration of iodinated contrast as part of semi-emergent coronary angiography for non-ST elevation ACS (NSTE-ACS). In addition to this, we are testing whether the levels of renal biomarkers are proportional to the increase in creatinine arising from the administration of iodinated contrast and can therefore predict the occurrence of CIN.

### Aims

The main aim of this project is to improve renal outcomes after angiography for NSTE-ACS, determining whether CIN can be predicted using novel biomarkers and prevented by prior treatment with inorganic NO_3_^−^.

### Study Design

This is a randomized, single-center, double-blind placebo-controlled trial testing the impact of potassium nitrate (KNO_3_) capsules versus potassium chloride (KCl) capsule placebo control.

### Study Population

Patients presenting with NSTE-ACS undergoing invasive coronary procedures that involve the administration of intracoronary iodinated contrast media (angiography +/-PCI) that require CIN prophylaxis will be recruited to the study. These patients will be recruited at The Barts Heart Centre, based at St Bartholomew’s Hospital. This is the largest cardiac center in the UK, serving a population of approximately 6 million people from North East and Central London and is a 24/7 center performing approximately 6000 angiograms and 2000 non-primary angioplasties a year. A total of 640 patients (male and female, aged ≥18) undergoing angiography for NSTE-ACS who require CIN prophylaxis as per local guidelines will be recruited. See Table 1 for full inclusion and exclusion criteria.

**Table 1.** Inclusion and Exclusion Criteria of NITRATE-CIN Study.

**Inclusion Criteria** Patients undergoing coronary angiography+/-PCI for NSTE-ACS Requirement for CIN prophylaxis as per Barts Heart Centre Criteria for CIN prophylaxis: ○ eGFR <60 mL/min **OR** ○ 2 of the following: diabetes, liver failure (cirrhosis), age > 70 yr, exposure to contrast in last 7 days, heart failure (or LVEF < 40%), concomitant renally active drugs (ACEi, ARB, NSAIDs, aminoglycosides, diuretics) Aged ≥18 Patients able and willing to give their written informed consent. **Exclusion Criteria** ST segment elevation myocardial infarction undergoing Primary PCI. Patients with eGFR<20 mL/min or on renal replacement therapy Subjects presenting with cardiogenic shock (systolic blood pressure <80 mmHg for >30 minutes, or requiring inotropes or emergency intra aortic balloon pump for hypotension treatment) or cardiopulmonary resuscitation Current life-threatening condition other than vascular disease that may prevent a subject completing the study. Use of an investigational device or investigational drug within 30 days or 5 half-lives (whichever is the longer) preceding the first dose of study medication. Patients considered unsuitable to participate by the research team (e.g., due to medical reasons, laboratory abnormalities, or subject’s unwillingness to comply with all study related procedures). Severe acute infection Pregnancy. This will be tested by urine HcG measurement Any Inclusion Criteria not met

### Intervention

Patients will be randomized to receive KNO_3_ capsules (12 mmol giving 744 mg of NO_3_^−^) or an equivalent dose (12 mmol) of KCl as placebo control. The capsules will be taken by the patient prior to their coronary angiogram procedure and they will receive a daily dose for 4 days post procedure (5 doses in total). A previous study from our group^20^ confirms tolerability of KNO_3_ with minimal side effects. In this study, we also demonstrated that the levels of circulating NO_2_^−^ achieved with a 12 mmol KNO_3_ dose were approximately 1.0 µmol/L, a level that persisted for up to 3 hours following ingestion. In contrast, a lower dose of 4 mmol did not achieve these levels (rising to no more than 0.5 µmol/L). In the NITRITE AMI trial, where a reduction of CIN events appeared to occur, the levels of circulating NO_2_^−^ were 0.67 ± 0.18 µmol/L.^17^ Thus, we used a 12 mmol dose for this study to ensure sufficient efficacious levels of the anion are achieved. The KNO_3_ and matching KCl placebo capsules will be supplied by the Pharmacy Manufacturing Unit based at Guy’s and St Thomas’ NHS Foundation Trust (GSTT). GSTT Pharmaceuticals hold a license with the MHRA, which allows the manufacture, storage and distribution of a range of Sterile and Non Sterile Investigational Medical Products (IMPs) for Phase I, II, III and IV clinical trials.

### Randomization

Patients will be block randomized on a 1:1 basis to receive either KNO_3_ capsules or placebo KCl capsules using an algorithm written in a statistical package with a reliable pseudorandom number generator, and stratified by diabetes status. Block lengths will be varied randomly.

### Blinding

Treatment assignment in both the intervention and placebo groups will remain blinded until data lock and statistical analysis at the end of the study.

### Study Endpoints

The primary endpoints are:

1. Incidence of CIN, as defined by the Kidney Disease Improving Global Outcomes (KDIGO) criteria for acute kidney injury.^21^

The secondary endpoints are:

1. To determine if inorganic NO_3_^−^ reduces renal adverse events including long-term renal impairment caused by CIN, need for hemofiltration or hemodialysis.
2. To determine whether levels of serum biomarkers released secondary to renal injury can predict CIN.
3. To determine if inorganic NO_3_^−^ ingestion decreases renal injury as assessed by serum levels of renal biomarkers.
4. To determine if inorganic NO_3_^−^ ingestion decreases rates of post-procedural MI (Society for Cardiovascular Angiography and Intervention definition).
5. Assessment of cost effectiveness of inorganic NO_3_^−^ (Quality-adjusted Life Years [QALYs]) measured over 12 months of follow-up.
6. Assessment of cardiovascular endpoints (Major Adverse Cardiac Events [MACE]) including death, non-fatal MI, and unscheduled revascularization.

### Experimental Protocol

Patients will be approached on their arrival to Barts Heart Centre. Following consent, and prior to angiography, up to 40 mL of venous blood will be taken. This sample will be used to measure baseline renal function, inflammatory markers (including hs-CRP), renal biomarkers, and baseline plasma NO_3_^−^/ NO_2_^−^ (collectively referred to as NOx) levels. Where possible, urine and saliva samples will also be taken for analysis of NOx levels, renal biomarkers and NO_3_^−^ reducing bacteria.

The participant will then be randomized to daily ingestion of either KNO_3_ or KCl capsules (placebo). This will be commenced prior to their scheduled angiogram and continue daily (2 capsules) for 5 days. A quality of life assessment (EQ5D) will be performed prior to PCI (and study drug administration). EQ5D is an instrument, which evaluates the generic quality of life that was developed in Europe and is widely used. The EQ5D descriptive system is a preference-based HRQL measure with one question for each of the 5 dimensions that include mobility, self-care, usual activities, pain/discomfort, and anxiety/depression.

**Figure 1.**
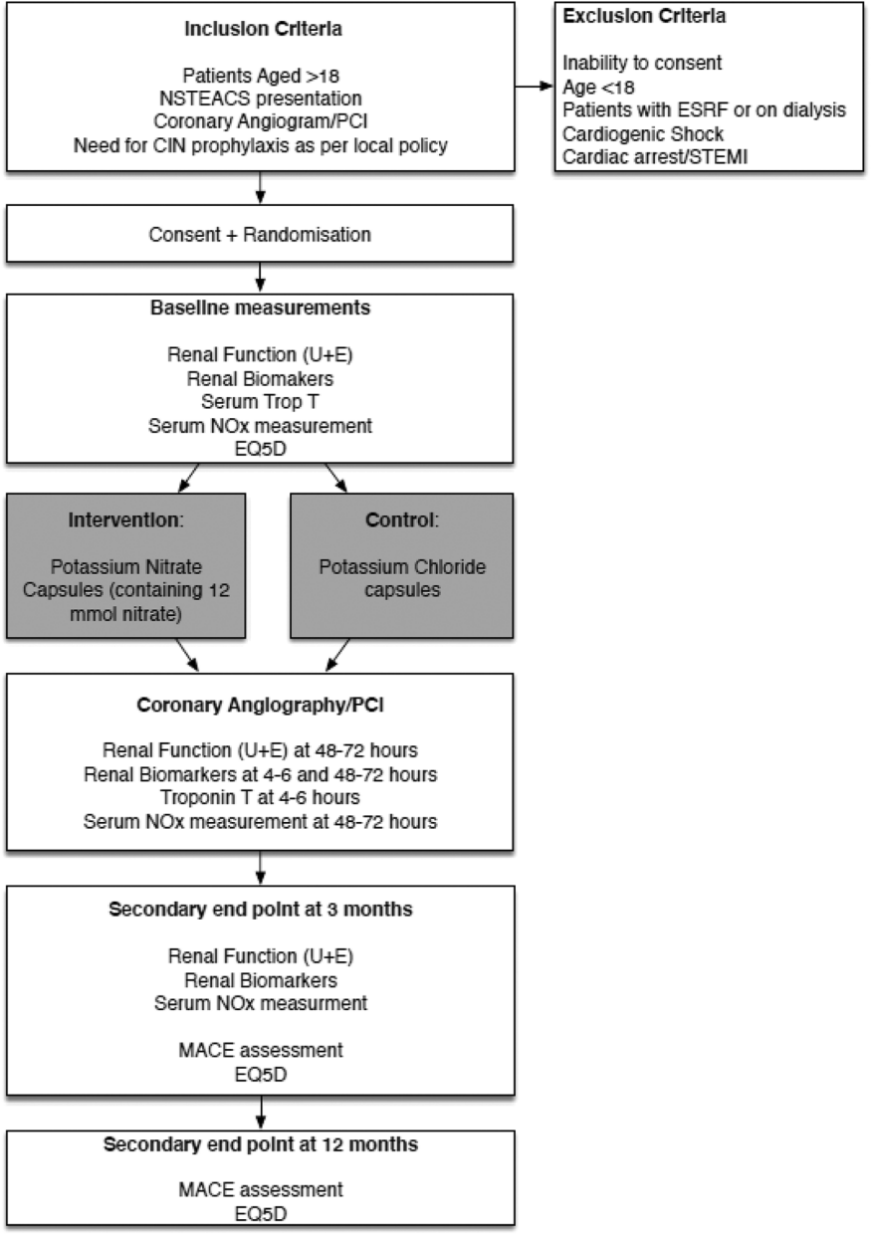
Study flowchart. NSTEACS: Non-ST segment elevation acute coronary syndrome; PCI: percutaneous intervention; CIN: contrast induced nephropathy; ESRF: end-stage renal failure; STEMI: ST segment elevation myocardial infarction; U&E: urea and electrolytes; NOx: nitric oxide levels (NO_2−_/ NO_3−_); MACE: major adverse cardiac events; EQ5D: quality of life questionnaire.

Following the angiogram +/− PCI procedure a blood sample will be collected 4-6 hours later for renal biomarkers, inflammatory markers and troponin (if PCI performed) assessment, along with urine and saliva sample collection. At 48-72 hours a blood sample for renal function will be taken, along with urine and saliva sample collection where possible. The participant will be asked to return to the clinical center at 3 months for review. At this stage venous blood and urine samples will be collected as well as EQ5D and assessment for MACE. After 1 year, the participant will be contacted by telephone for assessment of MACE and EQ5D. The whole duration of the study will be 1 year for each participant (see Figure 1). Participants have no obligation to complete the whole study and are free to withdraw at any point.

### Blood, Saliva and Urine Analysis

U&E and hs-CRP sample analysis will be performed at the local Barts Health Biochemistry laboratory. Blood samples will be taken from the venous side of the circulation from a hand vein using a yellow butterfly needle (19 gauge). Blood samples will be centrifuged immediately for plasma, and samples stored at −80 °C for the purposes of making biochemical measurements (e.g. NOx/cGMP). All samples will be discarded once used as per local procedures. Saliva and urine will be collected in a falcon tube at the time of visits and stored for analysis. Saliva will be centrifuged and a pellet generated. This pellet contains oral bacteria that have dislodged from the oral cavity. This pellet will be frozen (−80 °C) until identification of the oral microbiota by second-generation genome sequencing.

NOx concentrations in saliva, blood and urine will be determined using the technique of chemiluminescence, which will be performed at The William Harvey Research Institute (WHRI), Queen Mary University of London (QMUL) and measured at baseline, 4-6 hours, 48-72 hours, and at 3 months. Measures of renal biomarkers (serum and urine) will be conducted at WHRI (QMUL). These will also be measured at baseline, 4-6 hours, and at 48-72 hours.

### Management

This study is supported by the Barts Cardiovascular Clinical Trials Unit (CVCTU), a branch of the Barts CTU UKCRC Reg No. 4. The CVCTU will oversee the management and conduct of the trial, including safety reporting, coordination of trial committees, statistical analysis and reporting, and database management and electronic case report form (e-CRF) design.

Data will be captured in REDCap, a web-based electronic database, for all study participants and the database will be held on a secure server at QMUL. Participants eligible for the study will be given a screening number, and this number will be used to identify them throughout their study duration. The screening number will be identified on all e-CRFs and study documentation, e.g. questionnaires, lab reports. Only authorized users approved by the Chief Investigator (CI) will have access to the REDCap electronic database, and each user will be assigned specific user roles and rights. Sponsor representatives and CVCTU team members will have read-only access to the data. The study Research Nurse will be the primary person with delegated responsibility for data entry and eCRF completion.

### Adverse Events Reporting

All adverse events (AEs) and serious AEs will be recorded and reported to the principal investigator (PI). Related unexpected serious AEs, as assessed by the PI, will be reported within 24 hours of the research team becoming aware, to the Joint Research Management Office and the main Research Ethics Committee.

### Monitoring

A Trial Steering Committee composed of 3 independent experts in the fields of nephrology, interventional cardiology and clinical trials along with the investigators and 1-2 lay members will monitor the study. They will meet before patient recruitment and 6 monthly thereafter to assess trial conduct, recruitment, feasibility or other arising issues. An independent Data and Safety Monitoring Board (DSMB) will be formed to monitor patient safety. The DSMB will meet prior to initiation of the study, after recruitment of 10 patients, and thereafter at 6 monthly intervals. If there is serious concern with the safety of the participants the DSMB may recommend early termination of the study.

### Statistical Analysis

The reported incidence of ACS-associated AKI is extremely heterogeneous, ranging from 5% to 55%, and it varies with the criteria used for diagnosing AKI, the clinical setting and the investigated population.^4,5^ The most recent data from the Acute Coronary Treatment and Intervention Outcomes Network (ACTION) registry demonstrated that AKI occurred in 16% of 59 970 patients with acute myocardial infarction (AMI),^6^ with similar studies showing rates of 15% according to RIFLE criteria and 13% according to AKIN criteria in patients with AMI.^7^ These data were recently confirmed in the large-scale Harmonizing Outcomes With Revascularization and Stents in Acute Myocardial Infarction (HORIZONS-AMI) trial, which reported AKI in 16% of the patients.^8^ For our study calculations, we therefore propose a conservative CIN incidence of 12%. Our preliminary data^17^ demonstrated an 80%-related reduction in CIN. For our power calculations, we have proposed an effect at the lower end of this range and have powered the study to determine a difference of 60%. For a power of 80% and a significance level of 0.05, we need to recruit 232 patients into each trial arm, 464 patients in total, allowing for 27.5% drop out (primary endpoint) this means 320 patients in each arm and a total study population of 640 ACS patients. Subgroup analysis of the primary endpoint will be presented as unadjusted effect sizes by subgroup and for all patients using a modified forest plot, along with a test for heterogeneity. Subgroups will provide insight into the following mechanistic and exploratory questions.

1. **Pre-existing nitrate use versus no prior use:** Do patients already established on an organic nitrate derive similar benefits from inorganic nitrate therapy after an ACS? Previous studies have suggested less effect in patients established on organic nitrates.
2. **Diabetic versus non-diabetic patients:** Is dietary nitrate equally effective in diabetic and non-diabetic patients? Previous studies have suggested less benefit of nitrate in patients with diabetes.
3. **Troponin positive versus troponin negative**: Is the risk of CIN higher and dietary nitrate more effective in patients with evidence of myocardial infarct (troponin positive (NSTEMI) rather than troponin negative (unstable angina)).
4. **Mehran risk score group (low, moderate etc):** Is the benefit of inorganic nitrate seen throughout patients of varying CIN risk or is it only beneficial in those at the highest risk (Mehran score 11-15/ ≥16)?

### Economic Analysis

Economic evaluation will assist policy makers to decide whether inorganic NO_3_^−^ represents an efficient use of NHS resources. Cost-effectiveness analysis will be used to estimate the incremental costs and QALYs. Incremental cost per QALY gained by using inorganic NO_3_^−^ compared to standard care will be estimated by calculating the area under the curve for health utility using the EQ5D and health service costs up to 12 months. Quality of life and symptoms will be measured using the EQ5D questionnaires at baseline, 3 and 12 months after index admission. All health care consumption and costs will be estimated from a health system (NHS) perspective using patient self-reported questionnaires, and from hospital records. Costs will be attributed to the need for (i) continued hospitalization, (ii) procedural complications, and (iii) rehospitalization for renal dysfunction or myocardial ischemia. Costs of hospital admission will be measured using a top-down costing strategy. These costs will be measured for each patient in the trial and extrapolated to the general population to provide the estimated cost per patient.

## Summary

The NITRATE-CIN study is a proof-of- concept single-center, randomized, placebo-controlled, clinical trial designed to ascertain whether use of inorganic NO_3_^−^ might prove useful adjunctive therapy in improving renal function, reducing CIN and improving outcomes in ACS patients undergoing angiography +/− PCI.
